# Effects of β-blockers on all-cause mortality in patients with diabetes and coronary heart disease: A systematic review and meta-analysis

**DOI:** 10.3389/fcell.2023.1076107

**Published:** 2023-01-27

**Authors:** Shiqi Chen, Panhui Tian, Dannya Estau, Zijian Li

**Affiliations:** ^1^ Department of Cardiology and Institute of Vascular Medicine, Peking University Third Hospital, Beijing Key Laboratory of Cardiovascular Receptors Research, Key Laboratory of Cardiovascular Molecular Biology and Regulatory Peptides, Ministry of Health, Key Laboratory of Molecular Cardiovascular Sciences, Ministry of Education, Beijing, China; ^2^ Department of Pharmacy, Peking University Third Hospital, Beijing, China; ^3^ Department of Pharmacy Administration and Clinical Pharmacy, School of Pharmaceutical Sciences, Peking University, Beijing, China

**Keywords:** beta-blockers, coronary heart disease, diabetes mellitus, all-cause mortality, systematic review

## Abstract

Beta-blockers have been considered as an effective treatment in secondary prevention of coronary heart disease (CHD). However, there is still disputed whether β-blockers can increase all-cause mortality in patients with coronary heart disease and diabetes mellitus (DM). Here, our systematic review and meta-analysis is aiming to assess the effects of β-blockers on all-cause mortality in patients with coronary heart disease and diabetes mellitus. Four databases (PubMed, Embase, Cochrane Library and Web of Science) and other sources were searched to collect randomized controlled trials (RCTs) and cohort studies related to the treatment of β-blockers for coronary heart disease and diabetes mellitus patients. We further evaluated quality of evidence using the grading of recommendations assessment, development, and evaluation (GRADE) approach. Finally, a total of 16,188 records were identified, and four randomized controlled trials and six cohort studies (206,490 patients) were included. Random effects analysis revealed that β-blockers combined with routine treatment (RT) significantly decreased all-cause mortality in patients with coronary heart disease and diabetes mellitus compared with RT in control group (RR 0.59, 95% CI 0.47 to 0.75; *p* < 0.000 01; I^2^ = 72%). Subgroup analysis of all-cause mortality by the subtype of diabetes mellitus and definite MI patients (RR 0.54, 95% CI 0.45 to 0.65, *p* < 0.000 01, I^2^ = 29%) and the subtype of randomized controlled trials (RR 0.49, 95% CI 0.32 to 0.76, *p* = 0.001, I^2^ = 0%) indicated a relatively small heterogeneity and stable results. β-blockers application significantly reduced cardiovascular death as well (RR 0.56, 95% CI 0.42 to 0.74; *p* < 0.000 1; I^2^ = 0%). Our meta-analysis provided critical evidence of β-blockers treatment for patients with coronary heart disease (especially MI type) and diabetes mellitus, and discussed the advantages and potential metabolic risks for the clinical use of β-blockers. This study suggested that β-blockers application may improve all-cause mortality and cardiovascular death in coronary heart disease (especially MI type) and diabetes mellitus patients. However, given a small number of included studies, the aforementioned conclusion should be confirmed in a multi-center, large-scale, and strictly designed trial.

## 1 Introduction

Diabetes mellitus (DM) has long been treated as a comorbidity that affects the development and progression of coronary heart disease (CHD) (Arnold et al., 2020). Patients with DM increase the 2- to 4-fold risk of CHD, and around two-thirds of deaths are due to cardiovascular diseases (Goldfine and Beckman, 2008). Therefore, CHD patients with DM occupy a considerable proportion in the CHD population. In recent years, researchers have paid more attention to the potential treatment choices in the medical managements of CHD patients with DM (Arnold et al., 2020). Both secondary prevention strategies and glycemic managements play vital roles in the treatment of diseases. However, it is still unclear whether diabetic patients with CHD benefit from the combined managements (Fonseca, 2000; Cosentino et al., 2020).

Beta-adrenergic receptor blockers (β-blockers) have been advocated for the whole spectrum of CHD in guidelines and widely used in clinical medications in CHD patients (Ryden et al., 2013; Joseph et al., 2019). β-blockers can relieve symptoms of angina pectoris (Fox et al., 2006) and effectively improve the prognosis (reinfarction, sudden death and heart failure) in post-myocardial infarction (MI) patients with DM (Kjekshus et al., 1990). However, a prospective observational study has recently indicated that long-term β-blockers application may be associated with an increased risk of all-cause mortality for DM patients with CHD (Tsujimoto et al., 2017), as well as an increased risk for cardiovascular events in post-hoc analysis of a large-scale RCT (Tsujimoto et al., 2017). The rationality of β-blockers use in CHD patients with DM has been questioned and needs further assessment.

Here, we critically evaluated all-cause mortality and cardiovascular death of all the β-blockers in treating CHD combined with DM. A systematic review method was implemented by searching the databases, applying strict criteria, assessing the methodological quality, and evaluating outcomes.

## 2 Materials and methods

This review protocol was registered on PROSPERO (CRD 42022370904).

### 2.1 Criteria for considering the studies in this review

#### 2.1.1 Types of studies

Randomized controlled trials (RCTs) or cohort studies published in English.

#### 2.1.2 Types of participants

The patients that were identified according to CHD and diabetes diagnostic criteria, which were similar to previously published guidelines (Ryden et al., 2013; Cosentino et al., 2020), were eligible for inclusion in this study. Stable CHD, myocardial infarction (MI) or unstable angina pectoris combined with type 1 diabetes, type 2 diabetes or other types of diabetes were all included in the systematic review.

#### 2.1.3 Types of interventions in the experimental and control groups

The intervention in the experimental group included β-blockers and should be combined with the routine treatment (RT) of the control group. The intervention in the control group was the RT therapy without any β-blocker (non-users). RT should be the regular medications of glucose-lowering agents, lipid-lowering agents, antiplatelet and antithrombotic drugs as well as the management of arrhythmias and hypertension according to the guidelines (Ryden et al., 2013; Cosentino et al., 2020).

#### 2.1.4 Types of outcome measures

The primary outcome was defined as the measure of all-cause mortality. The additional outcomes observed included cardiovascular death and adverse effects.

### 2.2 Information sources and search strategy

The search was applied to the following four databases: PubMed, Embase, Cochrane Library and Web of Science, from the inception of each electronic database to 25 September 2022. Additional identification was conducted for all eligible trials by other searching methods from websites and citations. The following terms were used as the mesh terms or the free terms, for example, “diabetes mellitus”, “adrenergic beta-antagonist” and “β blocker.” The searching strategy in PubMed was performed as in Table 1 and detailed searching strategies were shown in Supplementary Table S1.

**TABLE 1:** Searching strategy in PubMed.

Step sequence	Details
#1	“diabetes mellitus” [MeSH Terms] OR [“diabetes” (All Fields) AND “mellitus” (All Fields)] OR “diabetes mellitus" [All Fields]
#2	(Adrenergic beta-Antagonist) OR (Adrenergic beta Antagonist) OR (β-blocker) OR (β blocker) OR (beta blocker) OR (adrenergic beta receptor blockader)
#3	Propranolol OR Labetalol OR Bisoprolol OR Metoprolol OR Arotinolol OR Nebivolol OR Esmolol OR Sotalol OR carteolol OR nadolol OR penbutolol OR pindolol OR timolol OR acebutolol OR atenolol OR betaxolol OR celiprolol OR bucindolol OR carvedilol OR alprenolol OR bunolol OR Bupranolol OR dihydroalprenolol OR iodocyanopindolol OR levobunolol OR metipranolol OR oxprenolol OR practolol
#4	#2 OR #3
#5	#1 AND #4

### 2.3 Study selection

Two investigators (SQC and PHT) independently performed a literature search according to the predetermined criteria in the Endnote 20 software. Initially, duplications were checked in all included databases and then removed from the original search results. Furthermore, the apparently irrelevant studies were excluded after reading the titles and abstracts. Finally, the unqualified studies were further excluded *via* screening the full-texts. The process of study selection was cross-checked by two researchers. Any disagreement in study selection was discussed and resolved in a consensus meeting with the corresponding author (ZJL).

### 2.4 Data extraction

After the selection, two authors (PHT and DNE) independently extracted data from the included studies *via* using a standardized sheet prepared for this review. The extracted data included the research title, year of publication, name of the first author, study types, disease types, sample size and interventions in the treatment and control groups, treatment duration or follow-up and outcome measures.

### 2.5 Risk of bias in individual studies

Two authors (PHT and DNE) independently assessed the quality of RCTs using assessment tools provided by the handbook of Cochrane Collaboration to evaluate the methodological quality of included studies, involving the blinding of outcomes assessment (i.e., detection bias), the blinding of participants and personnel (i.e., performance bias), the random sequence generation (i.e., selection bias), the allocation concealment (i.e., selection bias), the incomplete outcomes data (i.e., attrition bias), the selective reporting (i.e., reporting bias) and other biases. We also defined the following three situations as other biases: 1) whether the funder of the study has a stake in the outcome of the study, 2) whether the clinical trial was terminated early due to treatment benefit or side effects, and 3) whether the baseline is balanced for factors closely related to the outcome that we concerned. The quality of cohort studies was assessed *via* the Newcastle-Ottawa Scale (NOS), involving three aspects of selection (four points), comparability (two points) and outcome assessment (three points) for a total of nine points (Wells et al.). The high, moderate and low quality were scored as 7–9 points, 3–6 points and 0–3 points (Arab et al., 2019). Disagreements were resolved by consensus with the corresponding author (ZJL).

### 2.6 Strategy for data synthesis

#### 2.6.1 Statistical analysis

Review Manager 5.3 software provided by the Cochrane Collaboration was used to conduct data analysis. Dichotomous data of the outcome measures were calculated as the risk ratios (RR) and the 95% confidence interval (CI). *p* < 0.05 was considered to indicate a statistically significant difference.

#### 2.6.2 Assessment of heterogeneity

The heterogeneity of the included studies was analyzed with the χ2 test. When I^2^ ≤ 50%, a small heterogeneity was considered among the studies, and the fixed effects model was used for data analysis. In case the statistical heterogeneity was I^2^>50%, the random effects model was used and the sources of heterogeneity were measured. Subgroup analysis was performed in the presence of clinical heterogeneity, such as subtypes of CHD or different study types.

#### 2.6.3 Sensitivity analysis

The sensitivity analysis was performed with Stata software, version 13.0. Following the comparison of the pooled statistics after eliminating included studies one-by-one, certain differences could be found. We also conducted analysis by excluding RCTs with unclear risk of bias or low quality and cohort studies which were graded as low quality. Sensitivity analysis was also conducted to explore the stability of the results.

#### 2.6.4 Publication bias

Funnel plots were built to assess publication bias with more than 10 studies included. The Egger’s linear regression test and Begg’s test were used to further evaluate the symmetry of funnel plots. A qualified result of *p* > 0.05 in Egger’s and Begg’s tests indicated that no publication bias existed.

### 2.7 GRADE of evidence

Two authors (PHT and DNE) independently assess quality of evidence *via* grading of recommendations assessment, development, and evaluation (GRADE) system. GRADE can be classified as high, moderate, low, or very low qualities according to the judgment of the risk of bias, inconsistency, indirectness, imprecision, publication bias and other considerations. Summary of Findings (SOF) tables were produced by the online tool (GRADEpro GDT).

## 3 Results

### 3.1 Study selection

**FIGURE 1:**
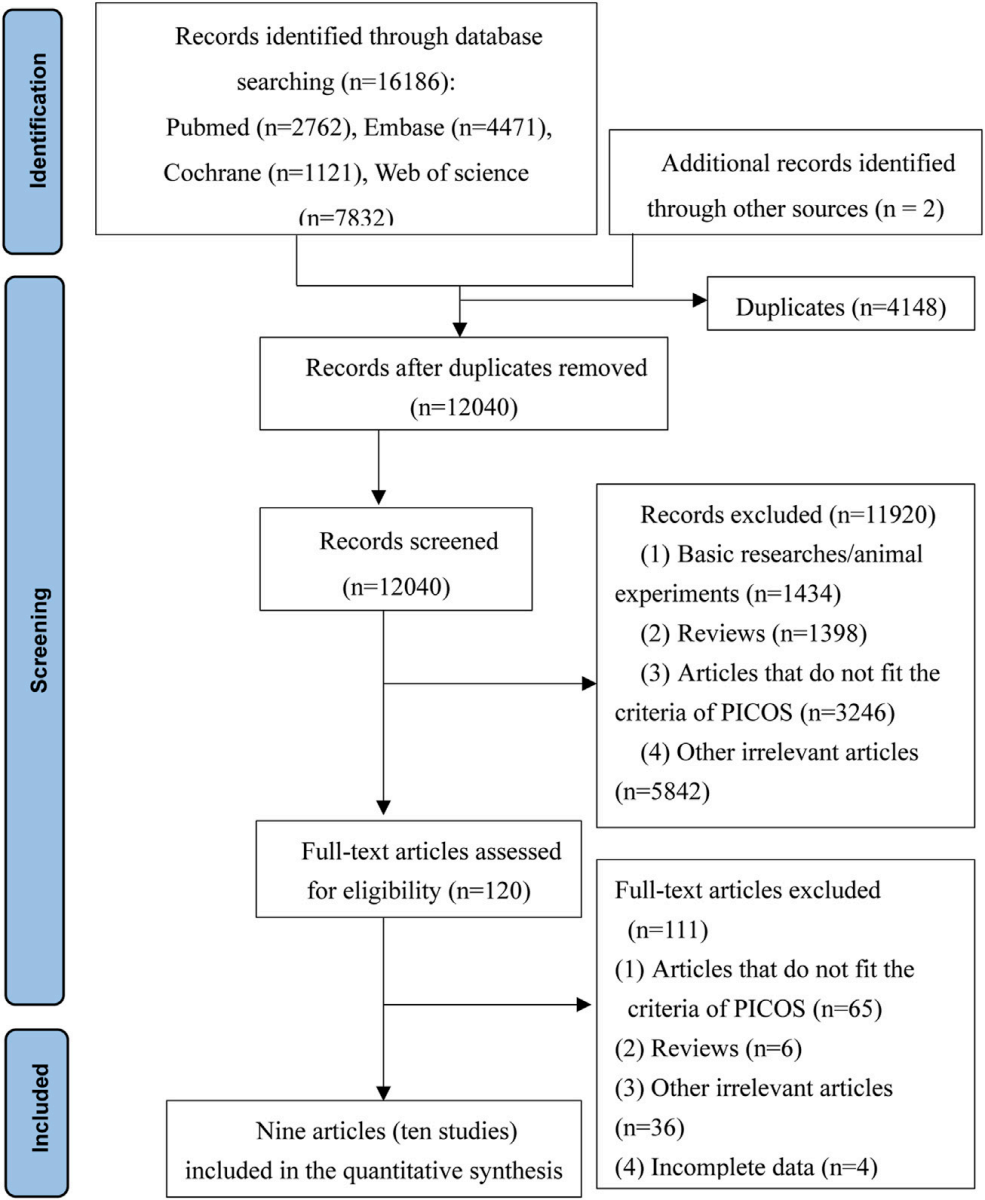
Flow chart for searching and screening of the articles.

A total of 1,6186 articles were retrieved from four electronic databases and two additional records were identified through other sources. After the removal of 4,148 duplicates, 12,040 potentially relevant articles remained for subsequent assessment. Following evaluating titles and abstracts, 11,920 articles were excluded. A total of 111 out of 120 remaining articles were excluded following the investigation of the full articles. Finally, nine publications including ten studies were included in the meta-analysis. A flow chart indicated the search process and study selection shown in Figure 1.

### 3.2 Study characteristics

A total of 16,186 records were identified and nine articles (Group, 1983; Gundersen and Kjekshus, 1983; Malmberg et al., 1989; Kjekshus et al., 1990; Karlson et al., 1994; Jonas et al., 1996; Gottlieb et al., 1998; McDonald et al., 2005; Tsujimoto et al., 2018) were included, covering 206,490 participants. Two different studies (Malmberg and MIAMI studies) were involved in one article (Malmberg et al., 1989). All included studies were published from 1983 to 2018. Four RCTs (Group, 1983; Gundersen and Kjekshus, 1983; Malmberg et al., 1989) and six cohorts (Kjekshus et al., 1990; Karlson et al., 1994; Jonas et al., 1996; Gottlieb et al., 1998; McDonald et al., 2005; Tsujimoto et al., 2018) were included in the meta-analysis. The sample size of the included RCTs varied from 36 to 305 subjects, and the duration of β-blocker treatment (containing metoprolol, pindolol and timolol) ranged from 15 days to 2 years. The disease diagnoses of these four RCTs were definite MI combined with DM. In the cohort studies, the sample size varied from 70 to 82,752 subjects, and the applications of β-blocker were not specifically referred to any subtype. The max follow-up period ranged from 1 to 10 years, and two of the cohort studies (Karlson et al., 1994; Tsujimoto et al., 2018) included CHD/DM patients of all kinds, and patients in other four cohorts (Kjekshus et al., 1990; Jonas et al., 1996; Gottlieb et al., 1998; McDonald et al., 2005) were diagnosed with definite MI and DM. Further details regarding the characteristics of the included studies were shown in Table 2.

**TABLE 2:** Characteristics of the included studies.

Study ID	Type of study	Type of disease	Sample size (T/C)	Age (T/C)	Interventions			Duration/Follow-up (max)	Outcome measures
					Treatment group	Control group			
Malmberg et al. (1989)	RCT	MI + DM	29/49	NA	Metoprolol + RT		Placebo + RT	3 months	①
Malmberg et al. (1989)	RCT	MI + DM	141/164	NA	Metoprolol + RT		Placebo + RT	15 days	①
Group (1983)	RCT	MI + DM	22/14	NA	Pindolol + RT		Placebo + RT	2 years	①
Gundersen and Kjekshus (1983)	RCT	MI + DM	53/46	NA	Timolol + RT		Placebo + RT	17 months	①②③
Kjekshus et al. (1990)	Cohort	MI + DM	127/141	63 ± 9/65 ± 11	β-blocker + RT		RT	1 year	①②
Karlson et al. (1994)	Cohort	MI + DM	37/33	NA	β-blocker + RT		RT	1 year	①
Jonas et al. (1996)	Cohort	CHD + DM	911/1812	60 ± 7/60 ± 7	β-blocker + RT		RT	3 years	①②
Gottlieb et al. (1998)	Cohort	MI + DM	69,153/132,599	73.3 ± 8.8/74.5 ± 9.0	β-blocker + RT		RT	2 years	①
McDonald et al. (2005)	Cohort	MI + DM	298/327	66.9 ± 10.9/71.9 ± 10.8	β-blocker + RT		RT	9 years	①
Tsujimoto et al. (2018)	Cohort	CHD + DM	292/242	NA	β-blocker + RT		RT	10 years	①

①all-cause mortality; ②cardiovascular death; ③adverse effects. The numerical values of age are presented as mean value ±standard deviation in the treatment group (T) and the control group (C). Abbreviations: C, control group; CHD, coronary heart disease; DM, diabetes mellitus; MI, myocardial Infarction; NA, not available; RCT, randomized controlled trial; RT, routine treatment; T, treatment group.

### 3.3 Risk of bias and methodological quality

**FIGURE 2:**
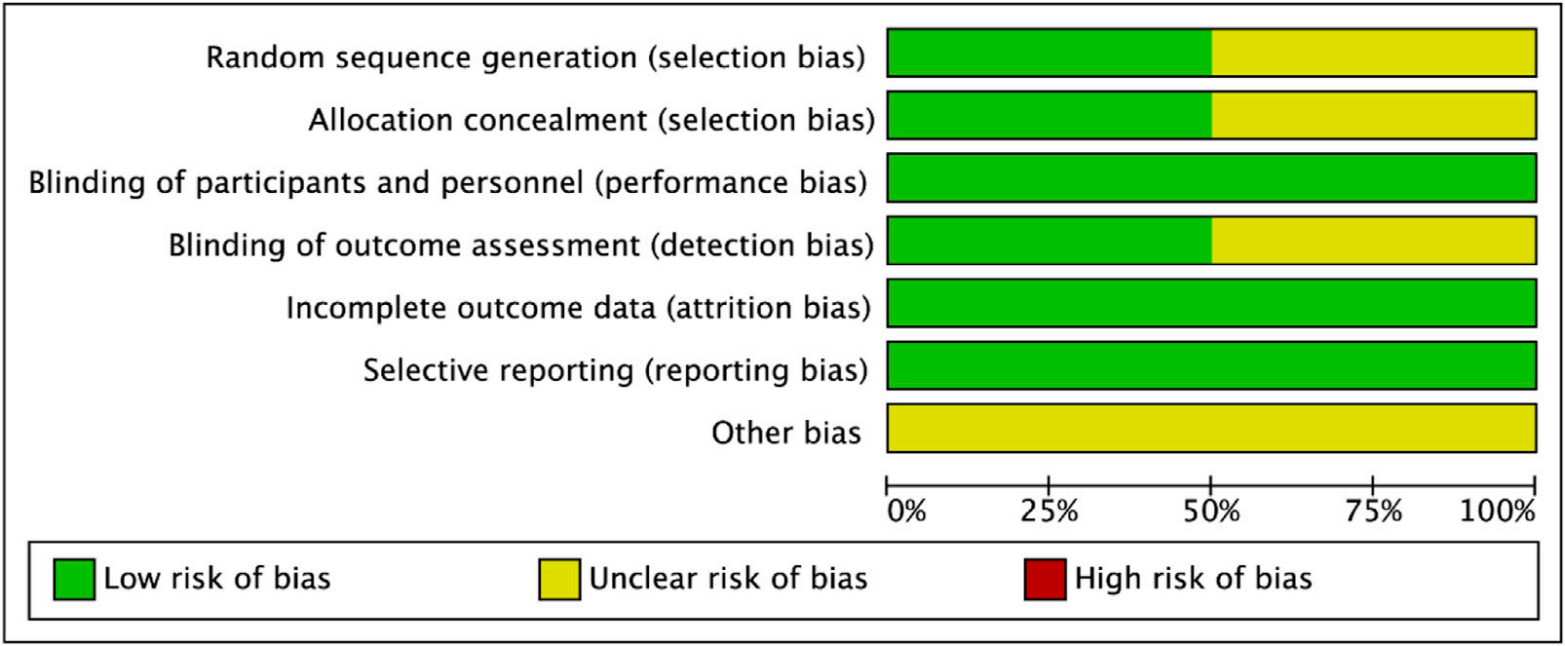
Risk of bias graph.

According to the assessment Cochrane ROB tool, the included four RCTs displayed methodological bias (Figure 2). All the included studies were described as “randomized” studies; two of them (Malmberg et al., 1989) reported using the “random number table.” The allocation concealment was also conducted in two studies (Malmberg et al., 1989), while the other two studies (Group, 1983; Gundersen and Kjekshus, 1983) were not clear. All of the studies reported the blinding of the participants and personnel, but only two studies reported the blinding of the outcome assessment (Malmberg et al., 1989). The data in the results were the same as the data in the original sources and no incomplete data were reported in the texts. Therefore, the incomplete outcome data were graded as low risks of bias. Also, the expected outcome indicators were reported and the selective reporting bias was defined as low risks of bias. We also evaluated the other biases, and the result showed that two of the included RCTs (Group, 1983; Gundersen and Kjekshus, 1983) did not specify the sources of funding and the other two (Malmberg et al., 1989) were funded by the pharmaceutical company unaware of the relationship with β-blockers. Therefore, we considered the other biases as unclear risk of bias.

Six cohort studies were assessed by NOS and the details were shown in Table 3. According to the scores, all cohort studies were graded as high qualities.

**TABLE 3:** The quality of cohort studies assessed *via* NOS.

Study ID	1.Selection				2.Comparability	3.Outcome			Total quality scores
	Representativeness of the exposed cohort	Selection of the non-exposed cohort	Ascertainment of the exposure	Demonstration that outcome of interest was not present at start of study	Comparability of cohorts on the basis of the design or analysis	Assessment of the outcome	Was follow-up long enough for outcomes to occur	Adequacy of follow up of cohorts	
Kjekshus (1990)	*	*	*	*	**	*	*	*	9
Karlson (1994)	*	*	*	—	**	*	*	*	8
Jonas (1996)	—	*	*	—	**	*	*	*	8
Gottlieb (1998)	*	*	*	—	**	*	*	*	8
McDonald (2005)	*	*	*	—	**	*	*	*	8
Tsujimoto (2018)	*	*	*	*	**	*	*	*	9

Indicates 1 points; ** indicates 2 points.

### 3.4 Outcome measures

#### 3.4.1 All-cause mortality of disease subtypes

**FIGURE 3:**
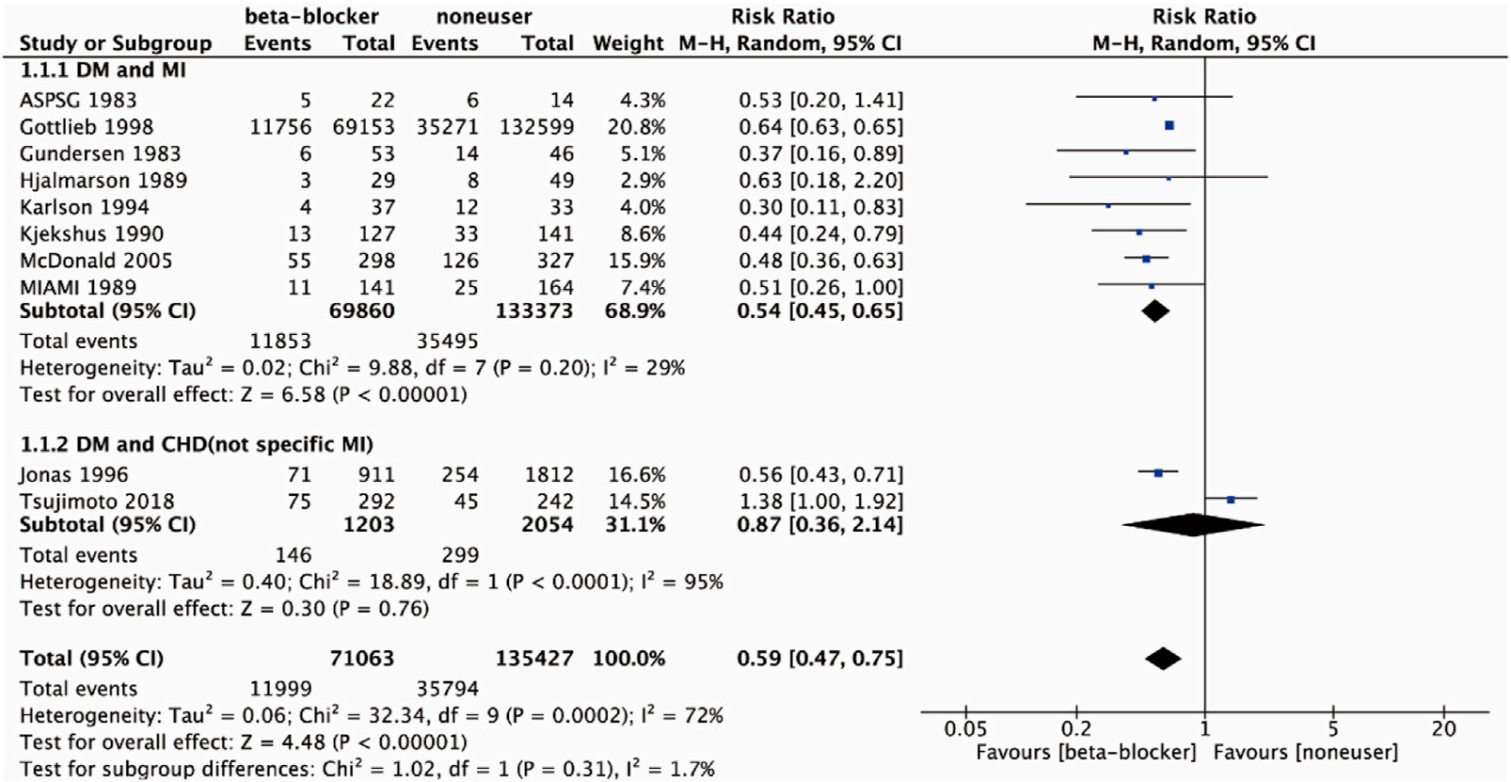
All-cause mortality of the included studies in disease subtypes.

In total, ten studies reported the outcome of all-cause mortality. Eight studies (Group, 1983; Gundersen and Kjekshus, 1983; Malmberg et al., 1989; Kjekshus et al., 1990; Karlson et al., 1994; Gottlieb et al., 1998; McDonald et al., 2005) included MI and DM patients, two studies (Jonas et al., 1996; Tsujimoto et al., 2018) included patients with DM and CHD, which were not specifically referred to MI. Subgroup meta-analysis was performed due to the varied disease subtypes and a large total heterogeneity (I^2^ = 72%). The random effects model was used in the meta-analysis. As shown in Figure 3, the combination of β-blockers with RT therapy performed better in reducing all-cause mortality in patients with DM and CHD (RR 0.59, 95% CI 0.47 to 0.75; *p* < 0.000 01; I^2^ = 72%). Results of different subgroups showed β-blockers with RT therapy in DM and MI patients (RR 0.54, 95% CI 0.45 to 0.65; *p* < 0.000 01; I^2^ = 29%) and in DM and CHD (not specific MI) patients (RR 0.87, 95% CI 0.36 to 2.14; *p* = 0.76; I^2^ = 95%). Therefore, it could be used to improve all-cause mortality for DM and CHD, and the heterogeneity among DM and MI subgroup reduced to 29%.

#### 3.4.2 All-cause mortality of study types

**FIGURE 4:**
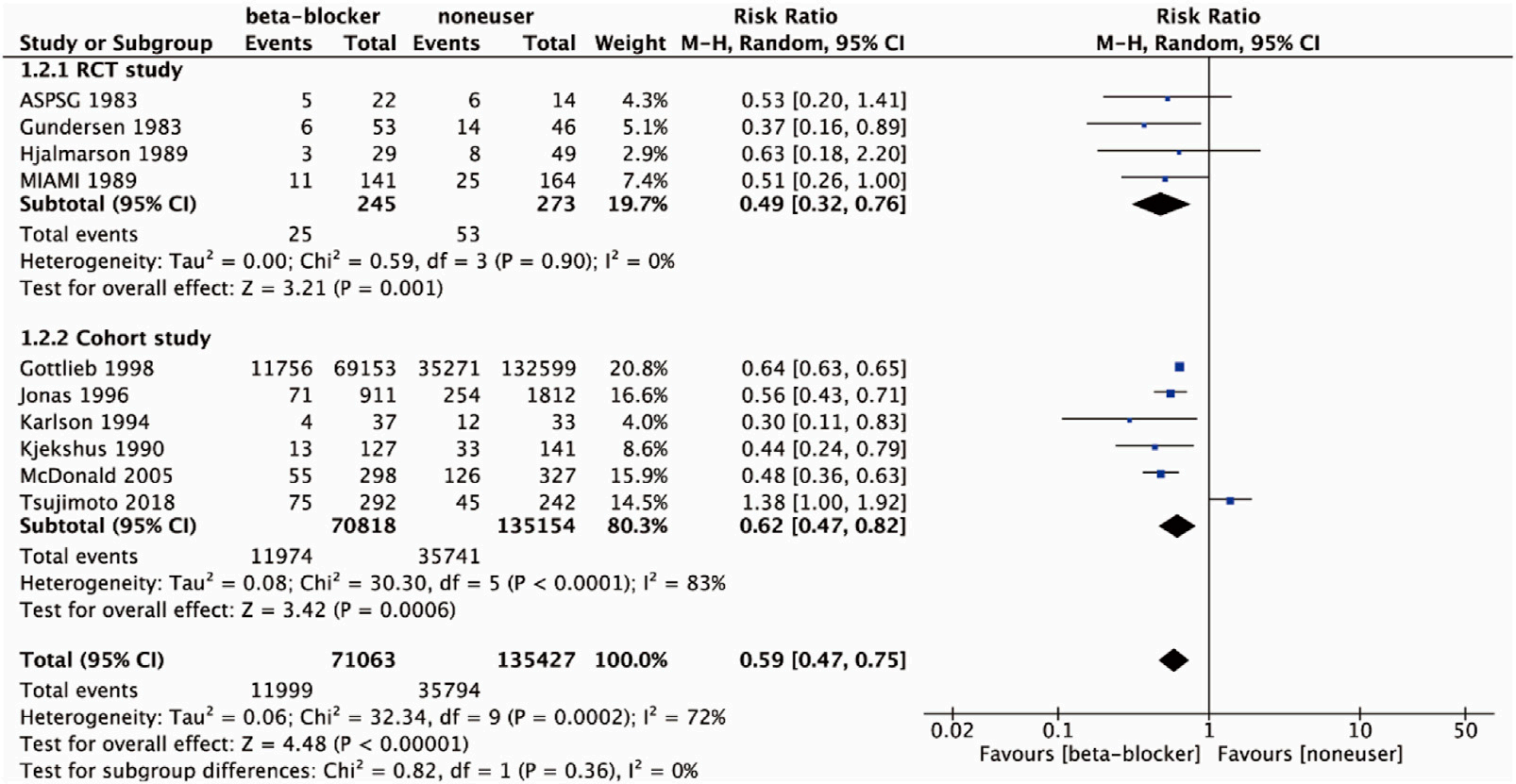
All-cause mortality of the included studies in study types.

Four studies (Group, 1983; Gundersen and Kjekshus, 1983; Malmberg et al., 1989) were RCTs, and six studies (Kjekshus et al., 1990; Karlson et al., 1994; Jonas et al., 1996; Gottlieb et al., 1998; McDonald et al., 2005; Tsujimoto et al., 2018) were cohort studies. Subgroup meta-analysis was performed due to the study types and a large total heterogeneity (I^2^ = 72%). The random effects model was used in the meta-analysis. Results of different subgroups showed β-blockers with RT therapy in RCTs (RR 0.49, 95% CI 0.32 to 0.76; *p* = 0.001; I^2^ = 0%) and cohort studies (RR 0.62, 95% CI 0.47 to 0.82; *p* = 0.000 6; I^2^ = 83%), as shown in Figure 4. Therefore, it could be used to improve all-cause mortality for DM and CHD of all the study types, and the heterogeneity among RCT subgroup reduced to 0%.

#### 3.4.3 Cardiovascular death

**FIGURE 5:**
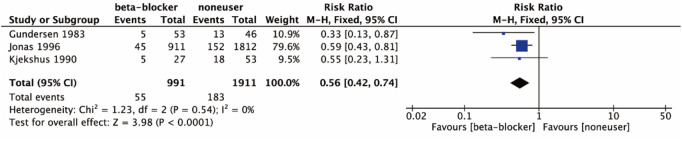
Cardiovascular death of the included studies.

Three studies (Gundersen and Kjekshus, 1983; Kjekshus et al., 1990; Jonas et al., 1996) reported cardiovascular death. As shown in Figure 5, the results indicated that β-blocker with RT therapy caused a significant decrease in cardiovascular death (RR 0.56, 95% CI 0.42 to 0.74; *p* < 0.0001; I^2^ = 0%).

#### 3.4.4 Adverse effects

One study (Gundersen and Kjekshus, 1983) reported adverse effects both in timolol treatment group and placebo group. Three heart failure cases, one atrioventricular block (II-III) case, six cold hands and feet cases, two cerebrovascular cases and three claudication cases were reported in timolol treatment group. One heart failure case, two atrioventricular block (II-III) cases, two hypoglycemia cases, three cerebrovascular cases and two claudication cases were reported in placebo control group. Further details were warranted to perform the assessment of causality and severity of the adverse effects.

#### 3.4.5 Sensitivity analysis

**FIGURE 6:**
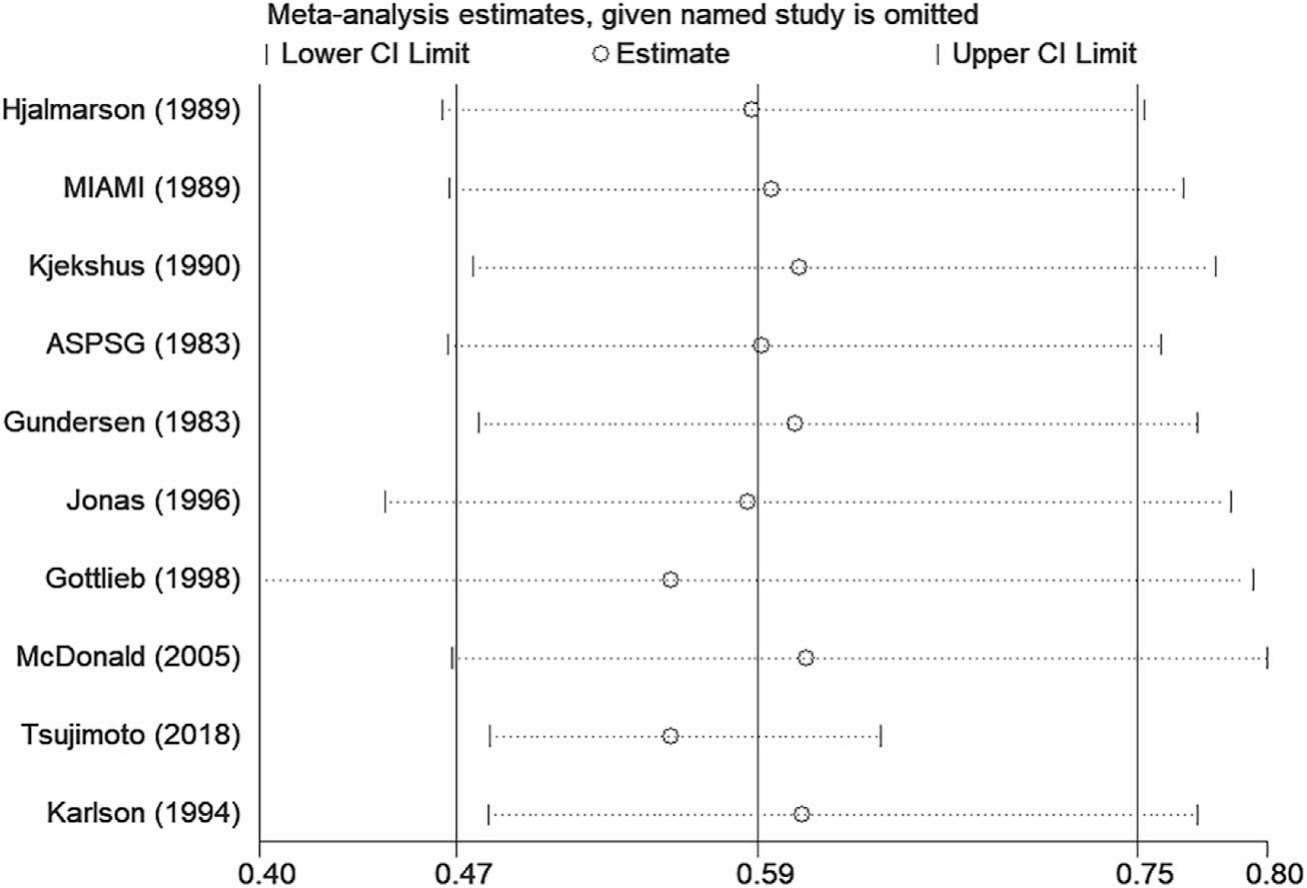
Sensitivity analysis in all-cause mortality.

The corresponding pooled all-cause mortality varied from 0.56 (0.40, 0.79) [excluding Gottlieb 1998 (Gottlieb et al., 1998)] to 0.61 (0.47, 0.80) [excluding McDonald 2005 (McDonald et al., 2005)]. Results were shown in Figure 6. After excluding RCTs (Group, 1983; Gundersen and Kjekshus, 1983) with unclear risks of randomization, allocation concealment and blinding of outcome assessment, the result of all-cause mortality was also stable [0.56 (0.39, 0.81)]. After excluding one cohort study (Tsujimoto et al., 2018) with significantly different findings from others, the result of all-cause mortality was also stable [0.56 (0.49, 0.64)], but the heterogeneity reduced from 72% to 27%, which showed the source of heterogeneity. After sensitivity analysis, the results did not influence the stability of the overall all-cause mortality 0.59 (0.47, 0.75) estimated in this meta-analysis.

#### 3.4.6 Publication bias

Due to a small number of four RCTs and six cohort studies included in our analysis, the funnel plot failed to accurately reflect the asymmetry and was not used to illustrate publication bias. The results of the Begg’s test (*p* = 1.000) and the Egger’s test (*p* = 0.564) demonstrated no evidence of significant publication bias. In general, considering only ten included studies, the results of publication bias may be not stable.

#### 3.4.7 GRADE

The quality of evidence was evaluated with GRADE system, and most of the outcomes were considered as the low score. The outcomes exhibited the low or very low evidence due to the unspecific risk of biases, the large heterogeneity, the wide confidence interval, low sample size and/or potential publication bias, as shown in Table 4.

**TABLE 4:** GRADE quality of evidence summary table.

Outcomes	Anticipated absolute effects^a^ (95% CI)		Relative effect (95% CI)	№ of participants (studies)	Certainty of the evidence (GRADE)	Comments
	Risk with placebo	Risk with β-blockers				
All-cause mortality (for RCTs)	194 per 1,000	95 per 1,000 (62–148)	RR 0.49 (0.32–0.76)	518 (4 RCTs)	⊕⊕○○ Low^b^ ^,^ ^c^	
All-cause mortality (for cohort studies)	264 per 1,000	164 per 1,000 (124–217)	RR 0.62 (0.47–0.82)	205,972 (6 observational studies)	⊕⊕○○ Low^d^	
Cardiovascular death (for RCT)	283 per 1,000	93 per 1,000 (37–246)	RR 0.33 (0.13–0.87)	99 (1 RCT)	⊕○○○ Very low^e^ ^,^ ^f^ ^,^ ^g^	
Cardiovascular death (for cohort studies)	91 per 1,000	53 per 1,000 (39–72)	RR 0.58 (0.43–0.79)	2,803 (2 observational studies)	⊕⊕○○ Low^h^	

^a^ The risk in the intervention group (and its 95% confidence interval) is based on the assumed risk in the comparison group and the relative effect of the intervention (and its 95% CI).CI: confidence interval; RR: risk ratio. GRADE Working Group grades of evidenceHigh certainty: we are very confident that the true effect lies close to that of the estimate of the effect. Moderate certainty: we are moderately confident in the effect estimate: the true effect is likely to be close to the estimate of the effect, but there is a possibility that it is substantially different. Low certainty: our confidence in the effect estimate is limited: the true effect may be substantially different from the estimate of the effect. Very low certainty: we have very little confidence in the effect estimate: the true effect is likely to be substantially different from the estimate of effect.

^b^ The random sequence generation and allocation concealment of some included studies were not specified.

^c^ The confidence interval is wide.

^d^ The results of Tsujimoto’s 2018 study went in a different direction to the results of other studies and I^2^ of 83% showed the inconsistence among studies.

^e^ The random sequence generation and allocation concealment of this study were not specified.

^f^ The number of patients was insufficient.

^g^ Only 1 study was included, so we strongly suspect that there may have publication bias.

^h^ Only 2 studies was included, so we strongly suspect that there may have publication bias.

## 4 Discussion

In the current systematic review and meta-analysis, we analyzed data of 206,490 patients from ten studies of nine publications, which indicated that β-blockers application induced all-cause mortality decreasing in patients with CHD and DM. We carried out the subgroup analysis of different disease types and research types, demonstrating that subgroups for DM and definite MI participants or RCTs had a robust outcome and a small heterogeneity for all-cause mortality decline. Furthermore, use of β-blockers in patients with CHD and DM can also reduce cardiovascular death. The adverse effects for timolol were described detailly in one study (Gundersen and Kjekshus, 1983) but cannot be further assessed for their causality. Sensitivity analysis indicated that none of the included studies interfered with the stability of the combined all-cause mortality.

β-blockers have been recommended in recent guidelines as a routine management for chronic coronary syndromes (Knuuti et al., 2020) or chronic HF with reduced ejection fraction (Yancy et al., 2017). Previous studies have revealed that both early-use and long-term therapies of β-blockers significantly reduced reinfarction and death among post-MI patients (Andersson et al., 2014; Bangalore et al., 2014; Puymirat et al., 2016; Kim et al., 2020). In addition, specific β-blockers such as metoprolol achieved a reduction in reinfarction and ventricular fibrillation in acute MI (Chen et al., 2005). Evidence of β-blockers in favor of patients with CHD combined with DM has been obtained in this meta-analysis. The possible mechanisms of the effectiveness, the interpretation of the results and potential risks are discussed below.

β-blockers can block adrenergic beta receptor and act directly on human heart, leading to a reduction in heart rate, contractility and myocardial oxygen demand (Yoshikawa et al., 1996). As a result of the myocardial protective effects, β-blockers have been regarded as first-line therapy in angina and other ischemic heart diseases, and also identified effectively in CHD (especially MI type) and DM patients in this study. In addition to the different disease types and research types detected as the main sources of heterogeneity, we found that one cohort study (Tsujimoto et al., 2018) provided significant different findings from others, which showed the source of heterogeneity as well. Through reading this study (Tsujimoto et al., 2018), we detected that the included participants were diagnosed with CHD (the definition of a previous diagnosis of CHD, MI or angina pectoris in the original article) and DM, which were different from the other studies mainly in MI and DM patients. Considering the various subtypes of CHD, our systematic review may partially present the results of MI due to the limited number of included articles. It still needs further assessment in the whole CHD or other subtypes such as angina pectoris.

Commonly used β-blockers are classified into two types: non-vasodilating, selective β1-antagonists (e.g., metoprolol, bisoprolol and atenolol) and β-blockers with vasodilating properties (e.g., carvedilol, labetalol and nebivolol) (Toda, 2003). Selective β1-antagonists may have negative metabolic effects by increasing insulin resistance (Kovacic et al., 2008), weight gain (Leslie et al., 2007) and masking severe hypoglycaemic symptoms (Dungan et al., 2019), which is more likely to occur in concomitant diabetic patients. In DM patients with CHD or heart failure, the use of β-blockers was indicated the association with an increased risk for cardiovascular events, as well as the incidence of severe hypoglycemia (Tsujimoto et al., 2017). Previous study has also found that severe hypoglycemia was strongly associated with increased risks of adverse clinical outcomes including death in patients with type 2 diabetes (Zoungas et al., 2010). Although this systematic review has demonstrated that we can take advantage of the usage and achieve long-term survival benefits in CHD and concomitant DM patients, considering the negative metabolic side-effects of β-blockers, doctors and pharmacists should strengthen the monitoring of adverse drug reactions such as the hypoglycemia symptoms especially in diabetic patients.

The limitations of this systematic review could be summarized in the following four aspects: First of all, the number of studies included in the meta-analysis was considerably small, and only four RCTs and six cohort studies were involved in the meta-analysis. Moreover, the heterogeneity of all-cause mortality among all the CHD studies was relatively high. Due to a lower heterogeneity detected in the subgroup analysis, results of all-cause mortality may be more convincing in DM and MI patients. Patients in DM and CHD (not specific MI) showed a rather high heterogeneity, which may lead to unstable results and needs further assessment. Furthermore, only ten studies included in the meta-analysis may augment the publication bias. Finally, we predetermined the sources of heterogeneity from disease subtypes and research types in this review, with more studies added in the future, specific β-blocker types, different follow-up periods and multiple types of CHD should be considered for subgroup analysis.

## 5 Conclusion

In summary, the present meta-analysis demonstrated that β-blockers can be used to reduce all-cause mortality in patients with CHD and DM, especially in patients with MI and DM. Moreover, it may also ameliorate cardiovascular death. However, multicenter, large-scale, specific β-blocker application and strictly designed trials are still required to confirm these findings and identify the differences of β-blockers in clinical use. This systematic review has provided evidence for the usage of β-blockers in treating patients with CHD and DM.
