# Supplementary material for: Effects of β-blockers on all-cause mortality in patients with diabetes and coronary heart disease: A systematic review and meta-analysis
Source: Front Cell Dev Biol. 2023 Jan 27;11:1076107. doi: 10.3389/fcell.2023.1076107 (PMC9911879; doi:10.3389/fcell.2023.1076107)
Supplement: Supplementary file 1 [file Table1.docx]

**Search Strategies**

**Web of Science**

| **Steps** | **Search terms** | **Hits** |
| --- | --- | --- |
| **#1** | **(("diabetes" AND "mellitus") OR "diabetes mellitus") (topic)** | **858841** |
| **#2** | **(Adrenergic beta-Antagonist) OR (Adrenergic beta Antagonist) OR (β-blocker) OR (β blocker) OR (beta blocker) OR (adrenergic beta receptor blockades) OR (Propranolol OR Labetalol OR Bisoprolol OR Metoprolol OR Arotinolol OR Nebivolol OR Esmolol OR Sotalol OR carteolol OR nadolol OR penbutolol OR pindolol OR timolol OR acebutolol OR atenolol OR betaxolol OR celiprolol OR bucindolol OR carvedilol OR alprenolol OR butylol OR Bupranolol OR dihydroalprenolol OR iodocyanopinodolol OR levobunolol OR metipranolol OR oxprenolol OR practocyl) (topic)** | **209460** |
| **#3** | **#1 and #2** | **7832** |

**PubMed**

| **Steps** | **Search terms** | **Hits** |
| --- | --- | --- |
| **#1** | **"diabetes mellitus"[MeSH Terms] OR ("diabetes"[All Fields] AND "mellitus"[All Fields]) OR "diabetes mellitus"[All Fields]** | **575286** |
| **#2** | **(Adrenergic beta-Antagonist) OR (Adrenergic beta Antagonist) OR (β-blocker) OR (β blocker) OR (beta blocker) OR (adrenergic beta receptor blockader)** | **101785** |
| **#3** | **Propranolol OR Labetalol OR Bisoprolol OR Metoprolol OR Arotinolol OR Nebivolol OR Esmolol OR Sotalol OR carteolol OR nadolol OR penbutolol OR pindolol OR timolol OR acebutolol OR atenolol OR betaxolol OR celiprolol OR bucindolol OR carvedilol OR alprenolol OR bunolol OR Bupranolol OR dihydroalprenolol OR iodocyanopindolol OR levobunolol OR metipranolol OR oxprenolol OR practolol** | **83962** |
| **#4** | **#2 OR #3** | **118105** |
| **#5** | **#1 AND #4** | **2762** |

**Cochrane Library**

| **Steps** | **Search terms** | **Hits** |
| --- | --- | --- |
| **#1** | **MeSH descriptor: [Diabetes Mellitus] explode all trees** | **35692** |
| **#2** | **(("diabetes" AND "mellitus") OR "diabetes mellitus"):ti,ab,kw** | **73415** |
| **#3** | **#1 OR #2** | **77782** |
| **#4** | **MeSH descriptor: [Adrenergic beta-Antagonists] explode all trees** | **4644** |
| **#5** | **((Adrenergic beta-Antagonist) OR (Adrenergic beta Antagonist) OR (β-blocker) OR (β blocker) OR (beta blocker) OR (adrenergic beta receptor blockader)):ti,ab,kw (Word variations have been searched)** | **12734** |
| **#6** | **(Propranolol OR Labetalol OR Bisoprolol OR Metoprolol OR Arotinolol OR Nebivolol OR Esmolol OR Sotalol OR carteolol OR nadolol OR penbutolol OR pindolol OR timolol OR acebutolol OR atenolol OR betaxolol OR celiprolol OR bucindolol OR carvedilol OR alprenolol OR bunolol OR Bupranolol OR dihydroalprenolol OR iodocyanopindolol OR levobunolol OR metipranolol OR oxprenolol OR practolol):ti,ab,kw** | **18684** |
| **#7** | **#4 OR #5 OR #6** | **24661** |
| **#8** | **#3 AND #7** | **1121** |

**Embase**

| **Steps** | **Search terms** | **Hits** |
| --- | --- | --- |
| **#1** | **diabetes:ab,kw,ti OR (diabetes:ab,kw,ti AND mellitus:ab,kw,ti)** | **969,448** |
| **#2** | **'adrenergic beta antagonist':ab,kw,ti OR 'adrenergic beta-antagonist':ab,kw,ti OR**  **(adrenergic:ab,kw,ti AND 'beta antagonist':ab,kw,ti) OR (adrenergic:ab,kw,ti AND beta:ab,kw,ti AND antagonist:ab,kw,ti) OR (adrenergic:ab,kw,ti AND 'beta receptor':ab,kw,ti AND blockader:ab,kw,ti) OR 'β blocker':ab,kw,ti OR (β:ab,kw,ti AND blocker:ab,kw,ti) OR (beta:ab,kw,ti AND blocker:ab,kw,ti)** | **33,861** |
| **#3** | **propranolol:ab,ti,kw OR labetalol:ab,ti,kw OR bisoprolol:ab,ti,kw OR metoprolol:ab,ti,kw OR arotinolol:ab,ti,kw OR nebivolol:ab,ti,kw OR esmolol:ab,ti,kw OR sotalol:ab,ti,kw OR carteolol:ab,ti,kw OR nadolol:ab,ti,kw OR penbutolol:ab,ti,kw OR pindolol:ab,ti,kw OR timolol:ab,ti,kw OR acebutolol:ab,ti,kw OR atenolol:ab,ti,kw OR betaxolol:ab,ti,kw OR celiprolol:ab,ti,kw OR bucindolol:ab,ti,kw OR carvedilol:ab,ti,kw OR alprenolol:ab,ti,kw OR bunolol:ab,ti,kw OR bupranolol:ab,ti,kw OR dihydroalprenolol:ab,ti,kw OR iodocyanopindolol:ab,ti,kw OR levobunolol:ab,ti,kw OR metipranolol:ab,ti,kw OR oxprenolol:ab,ti,kw OR practolol:ab,ti,kw** | **89,702** |
| **#4** | **#2 OR #3** | **110,889** |
| **#5** | **#1 AND #4** | **4,471** |
